# Acute metabolic actions of the major polyphenols in chamomile: an *in vitro* mechanistic study on their potential to attenuate postprandial hyperglycaemia

**DOI:** 10.1038/s41598-018-23736-1

**Published:** 2018-04-03

**Authors:** Jose A. Villa-Rodriguez, Asimina Kerimi, Laszlo Abranko, Sarka Tumova, Lauren Ford, Richard S. Blackburn, Christopher Rayner, Gary Williamson

**Affiliations:** 1 School of Food Science and Nutrition, University of Leeds, Leeds, LS2 9JT UK; 2 School of Chemistry, University of Leeds, Leeds, LS2 9JT UK; 3 School of Design, University of Leeds, Leeds, LS2 9JT UK; 4 Present Address: Szent István University, Faculty of Food Science, Department of Applied Chemistry, 29-43 Villányi, Budapest, H-1118 Hungary

## Abstract

Transient hyperglycaemia is a risk factor for type 2 diabetes and endothelial dysfunction, especially in subjects with impaired glucose tolerance. Nutritional interventions and strategies for controlling postprandial overshoot of blood sugars are considered key in preventing progress to the disease state. We have identified apigenin-7-*O*-glucoside, apigenin, and (*Z*) and (*E*)−2-hydroxy-4-methoxycinnamic acid glucosides as the active (poly)phenols in Chamomile (*Matricaria recutita*) able to modulate carbohydrate digestion and absorption *in vitro* as assessed by inhibition of α-amylase and maltase activities. The latter two compounds previously mistakenly identified as ferulic acid hexosides were purified and characterised and studied for their contribution to the overall bioactivity of chamomile. Molecular docking studies revealed that apigenin and cinnamic acids present totally different poses in the active site of human α-amylase. In differentiated Caco-2/TC7 cell monolayers, apigenin-7-*O*-glucoside and apigenin strongly inhibited D-[U-^14^C]-glucose and D-[U-^14^C]-sucrose transport, and less effectively D-[U-^14^C]-fructose transport. Inhibition of D-[U-^14^C]-glucose transport by apigenin was stronger under Na^+^-depleted conditions, suggesting interaction with the GLUT2 transporter. Competitive binding studies with molecular probes indicate apigenin interacts primarily at the exofacial-binding site of GLUT2. Taken together, the individual components of Chamomile are promising agents for regulating carbohydrate digestion and sugar absorption at the site of the gastrointestinal tract.

## Introduction

Excess consumption of carbohydrates and sugars can lead to hyperglycaemia, endothelial dysfunction and insulin resistance, increasing the risk of developing of type 2 diabetes and cardiovascular diseases. The modulation of carbohydrate digestion in the intestine by the non-absorbed drug acarbose reduced the risk of type 2 diabetes and cardiovascular events by 25 and 49% respectively in a population with impaired glucose tolerant according to The Study To Prevent Non-Insulin dependent Diabetes Mellitus trial^1,2^, indicating the clinical relevance of this approach. Gastrointestinal side-effects of acarbose have resulted in poor patient compliance and adherence to the treatment, highlighting the need for the identification of new molecules which could restrict post-prandial burden. To this end, we previously screened several plant extracts and identified German chamomile (*Matricaria recutita*) (ChE) as very effective at attenuating carbohydrate digestion and sugar absorption *in vitro*^3^.

Chamomile is used as an herbal tea all over the world, and recognition of its medicinal properties is exemplified by its listing as an official drug in the pharmacopoeias of 26 countries including the UK^4^. It can potentially relieve the metabolic overload effects of high sugar diets such as glucose and fructose by attenuating their absorption through the inhibition of carbohydrate-digestive enzymes and apically-located transporters^3^; this mechanism may explain some of the positive effects observed by chamomile in experimental diabetes^5,6^ and type 2 diabetics^7,8^.

Chamomile contains many different phytochemicals, mainly (poly)phenols, a class of small molecules with several bioactivities^9,10^. (Poly)phenol content varies depending on growth conditions and extraction methods and this can have a pronounced impact on biological activities. Therefore, a first and essential step is to identify the specific active constituents responsible for the bioactivity. In another study where authors attempted to identify inhibitors of digestive enzymes in chamomile a convincing single candidate was not described^5^. Furthermore, individual components of chamomile have been previously reported to have a beneficial inhibitory effect on monosaccharide transport; however, the contribution of each on the overall exerted effect by ChE is not known. Here we report the inhibitory activity of the main (poly)phenol constituents of ChE on the different steps of carbohydrate digestion and absorption, while for the first time we have fully confirmed and characterized two previously misidentified glycosylated hydroxycinnamic acids present in ChE at high concentrations and assessed their contribution to the potential of ChE to counteract hyperglycaemia.

## Results

### Analytical and semi-preparative HPLC-DAD analysis of chamomile

**Figure 1:**
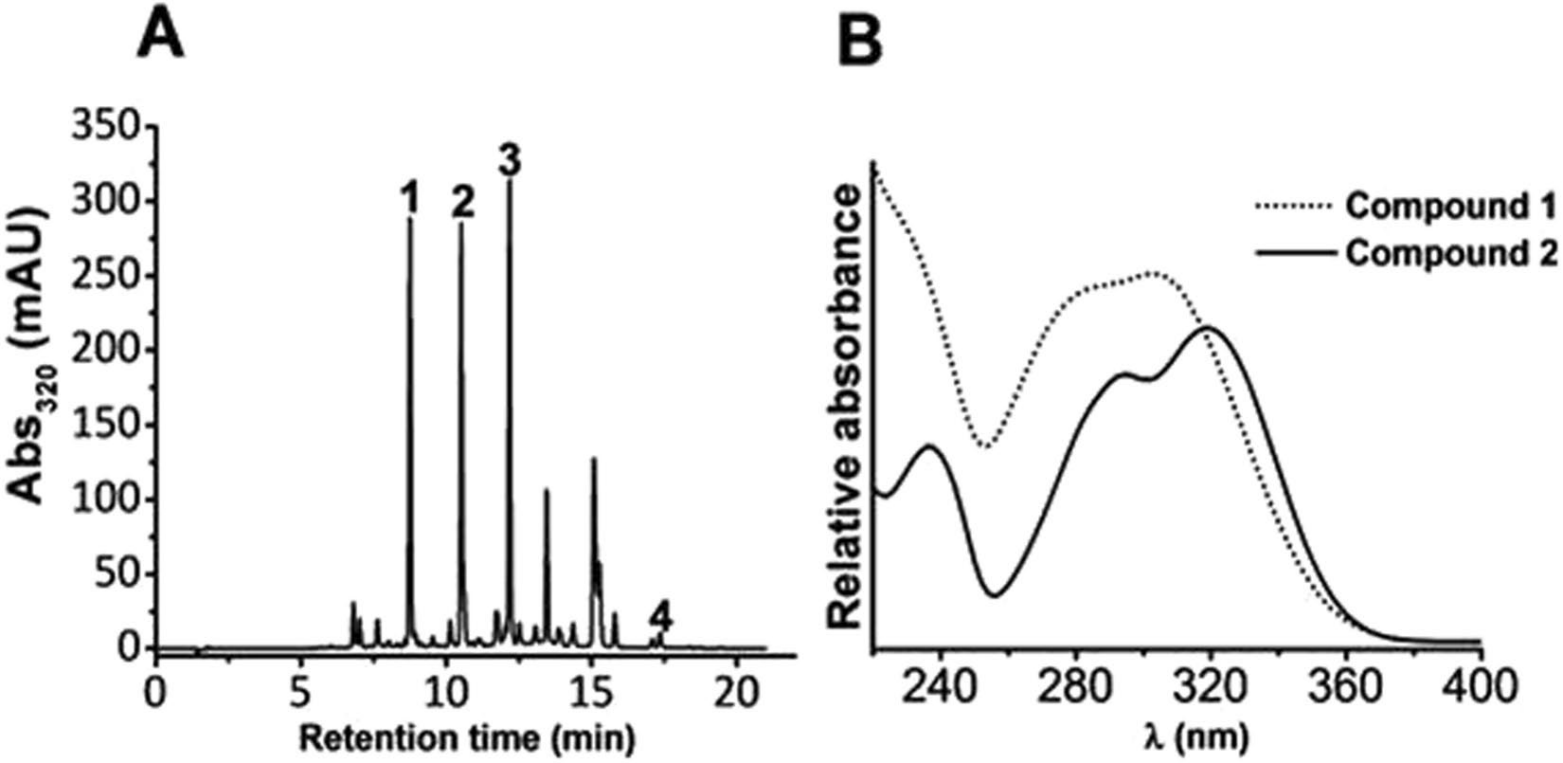
HPLC-DAD chromatogram of ChE extract. Targeted compounds are referred to according to the retention time sequence. For retention time, refer to Table 1.

Initial detection of target (poly)phenols in ChE was based on our previous report^3^ where we identified by mass spectrometry apigenin, apigenin-7-*O*-glucoside and two compounds with corresponding [M-H]^-^ ion masses at *m/z* 355 and 711 (compounds 1 and 2) as the most abundant constituents. In this study, apigenin-7-*O*-glucoside (compound 3) and apigenin (compound 4) (Fig. 1A; Table 1) were detected based on the comparison of their retention time with those of authentic standards. The UV absorption spectra of compounds 1 and 2 (Fig. 1B) were characteristic of methoxycinnamic acid derivatives. The compounds were separated, isolated and concentrated by semi-preparative column chromatography (supplementary S1) yielding enough material to perform *in vitro* experiments. The purity of the collected fractions, pooled after freeze-drying, was >80% for compound 1 and >99% for compound 2 as assessed by liquid chromatography-mass spectrometry (LC-MS) based on the total ion count chromatograms (TIC).

**Table 1:** Identification and quantification of targeted (poly)phenols in ChE.

Compound	Rt (min)	Concentration (µM)	LOD (µM)	LOQ (µM)
1*	8.7	257.0 ± 2.2	0.42	1.32
2	10.5	200.9 ± 2.0	0.46	1.43
3	12.1	148.7 ± 2.0	2.62	8.18
4	15.8	12.22 ± 0.08	2.48	7.75

*The quantification was conducted with the isolated standard with a purity ≥80%. An isomerisation reaction took place where (*Z*)-MCAG (compound 1) changed to the most thermodynamically stable form (*E*)-MCAG (compound 2) which accounts for the impurity detected. The identification of compound 1 and 2 is described in section 3.2.

### Chemical characterisation of the hydroxycinnamic acid derivatives

The identities of the hydroxycinnamic acid derivatives were assigned based on HPLC-DAD/QTOFMS, ^1^H and ^13^C NMR, NOESY and chemical synthesis. The full-scan TOF spectra of both compounds showed prominent ions at *m/z* 355.1035 and 711.2136, which were assigned as [M-H]^-^ and [2M-H]^-^ respectively (Table 2). This assignment was further confirmed by the appearance of the [M+Cl]^-^ ion at *m/z* 391.0801 in the spectrum of compound 2. The (neutral) elemental formula of the two hydroxycinnamic acid derivatives was established as C_16_H_20_O_9_. Both compounds showed identical fragment ions in the Q/TOF product ion spectra at *m/z* 193.0506 [M-hexosyl-H]^-^ and 149.0608 [M-hexosyl-CO_2_]^-^. This result excluded the possibility that either of the compounds is a dimer of ferulic acid hexoside as suggested by Guimarães *et al*.^11^ based on the predominant ion at *m/z* 711 for compound 2. Covalently bound ‘dimeric ferulic acids’ are well characterised compounds, which act like polysaccharide cross-linking agents in cell walls^12,13^, and therefore their (di)glycoside structures can also be theoretically expected in plant extracts. However, the predicted ion formula [C_32_H_37_O_18_]^-^ would result in a monoisotopic ion mass of *m/z* 709.1985 in negative ion mode. Confirmatory fragmentation of the *m/z* 711.2136 was performed by Q/TOF that provided fragments only at *m/z* 355 and 193, lending further support to our original assumption that the elemental formula of the two hydroxycinnamic acid derivatives is C_16_H_20_O_9_. Further analysis was performed to conclusively rule out the isomeric structures of ferulic acid hexosides previously suggested.^11,14,15^

**Table 2:** Q/TOF-MS/MS of isolated compounds from ChE.

Compound	[M-H]-	MS^2^	Elemental formula	Measured m/z	Exact mass	Error (ppm)
1	355	[M-hexosyl-CO_2_]-	C_9_H_9_O_2_	149.0599	149.0608	−6.04
	355	[M-hexosyl]-	C_10_H_9_O_4_	193.0496	193.0506	−5.18
	355	[M-H]-	C_16_H_19_O_9_	355.1017	355.1035	−5.07
	711	[M-hexosyl]-	C_10_H_9_O_4_	193.0497	193.0506	−4.66
	711	[M-H]-	C_16_H_19_O_9_	355.1029	355.1035	−1.69
	711	[2M-H]-	C_32_H_39_O_18_	711.2152	711.2136	2.25
2	355	[M-hexosyl-CO_2_]-	C_9_H_9_O_2_	149.0599	149.0608	−6.04
	355	[M-hexosyl]-	C_10_H_9_O_4_	193.0493	193.0506	−6.73
	355	[M-H]-	C_16_H_19_O_9_	355.1034	355.1035	−0.28
	711	[M-hexosyl]-	C_10_H_9_O_4_	193.0494	193.0506	−6.22
	711	[M-H]-	C_16_H_19_O_9_	355.1022	355.1035	−3.66
	711	[2M-H]-	C_32_H_39_O_18_	711.2114	711.2136	−3.09

**Figure 2:**
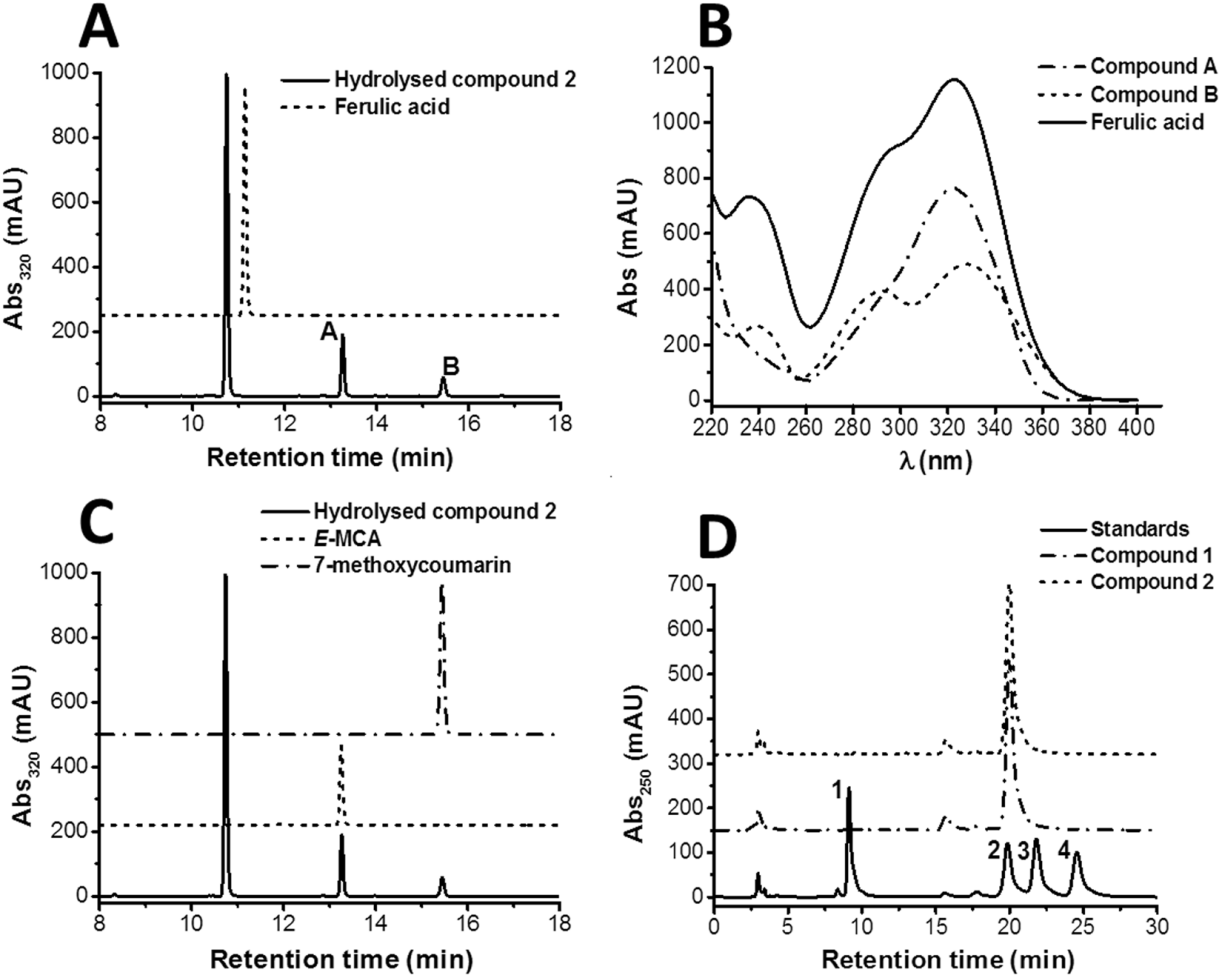
Analysis of compound 2 after acid hydrolysis. (**A**) HPLC-DAD chromatogram of the hydrolysed fraction and comparison of the released compounds with ferulic acid standard. (**B**) UV-Vis spectra of ferulic acid standard and compounds resulting from acid hydrolysis. (**C**) Comparison of the resulting compounds after acid hydrolysis with standards of (*E*)-MCA and 7-methoxycoumarin. (**D**) HPLC-DAD chromatograms of PMP derivatives of standard monosaccharides: 1-D-mannose; 2-D-glucose; 3-D-galactose; 4-D-xylose.

Acid hydrolysis of compound 2 did not result in the release of ferulic acid as would be expected if the compounds were ferulic acid hexosides (Fig. 2A–C). However, a compound with *m/z*^(-)^ 193 (supplementary S2) with UV spectra characteristic of hydroxycinnamates was detected at 13.4 min (Fig. 2C). We also observed a peak at 15.3 min with a UV spectra distinctive of coumarins^16^, later confirmed as 7-methoxycoumarin by comparison with the authentic standard (Fig. 2C). The presence of 7-methoxycoumarin was indicative of the release of a *O*-hydroxycinnamate which, under the acidic conditions^16^ employed for hydrolysis, underwent intramolecular cyclization.

**Figure 3:**
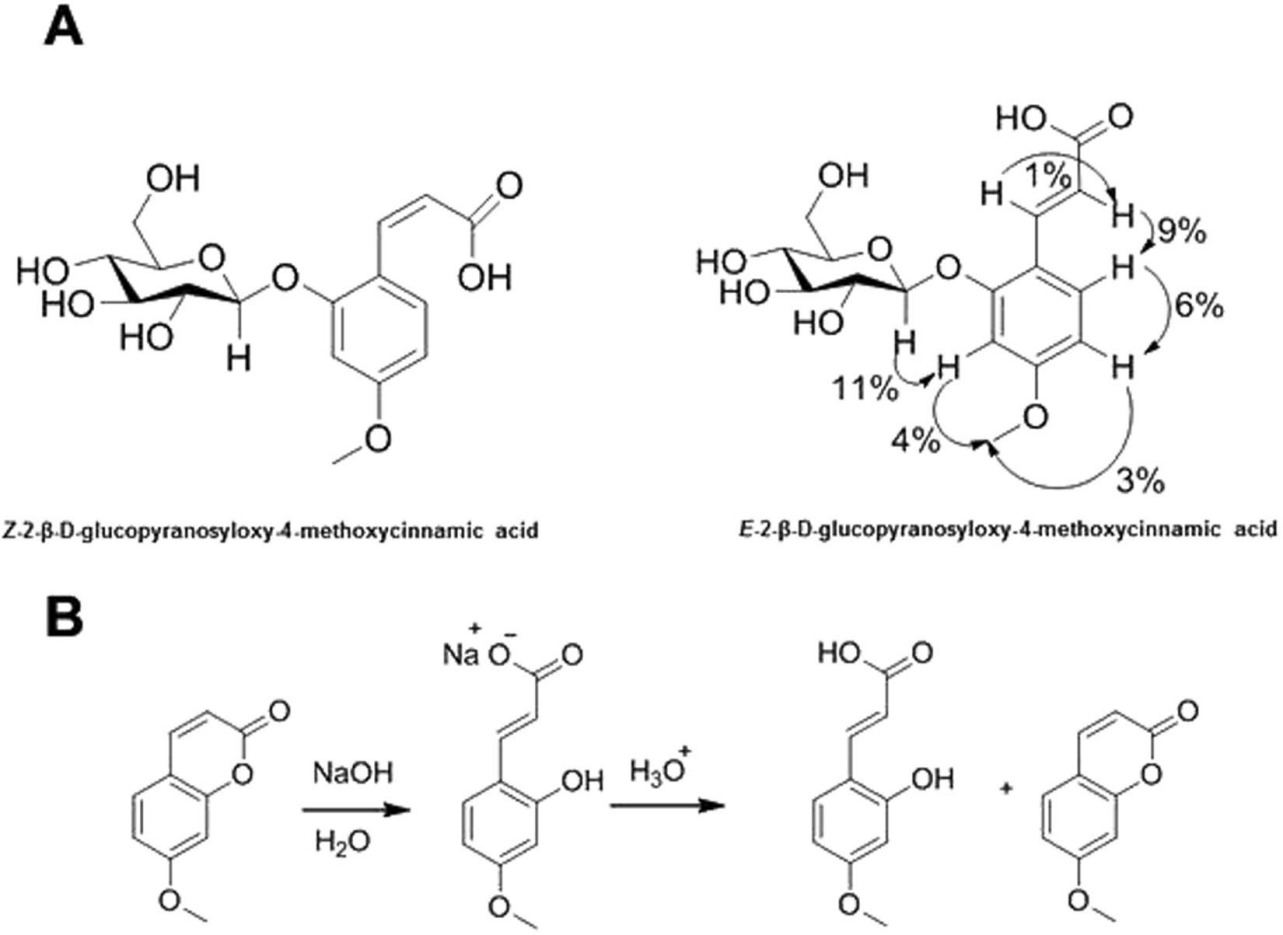
(**A**) Chemical structures of isolated compounds (1 & 2). Key NOESY correlations for compound 2 are indicated with arrows and percentage enhancements. (**B**) Scheme showing the synthetic aglycone and the corresponding coumarin ring closure.

The ^1^H-NMR data displayed spectroscopic signals characteristic of a free carboxylic group, which was identified from detection of the signal at δ 12.00. The presence of a (*Z*) and (*E*)-alkene group in the structure of compound 1 and 2 were identified from the signals at δ 7.19 and 5.78 (*J* = 12.7 Hz), and δ_H_ 7.82 and 6.39 (*J* = 16.2 Hz), respectively. The spectrum also indicated the presence of three aromatic protons in the ABD substitution pattern characteristic of the coupling constants of *J* = 8.5–8.6 (*ortho*) and *J* = 2.4–2.5 (*meta*)^17^. Acid hydrolysis of compounds 1 and 2 resulted in the release of D-glucose (Fig. 2D). The ^1^H chemical shift in the range 4.85 (*Z*-isomer) and 4.98 (*E*-isomer) with coupling constants of *J* = 7.5 Hz from the anomeric protons indicate a β-glycosidic linkage and the location of the D-glucose moiety was assigned at the aromatic ring by the observed NOE enhancements (Fig. 3A). The NOE enhancements gave an indication of the position of the other functional groups around the ring. The ^13^C NMR data showed 16 carbon resonances for the phenolic moiety in isolated compound 2, suggesting that the compounds are (*Z*) and (*E*)−2-β-D-glucopyranosyloxy-4-methoxycinnamic acid (*Z* and-*E*-MCAG). Further confirmation of the identity of the cinnamate moiety was obtained by chemical synthesis, and comparison with the released aglycone after acid hydrolysis (Fig. 2C).

### Inhibition of human α-amylase and rat α-glucosidase activities by ChE (poly)phenols

#### *In vitro* assays

**Figure 4:**
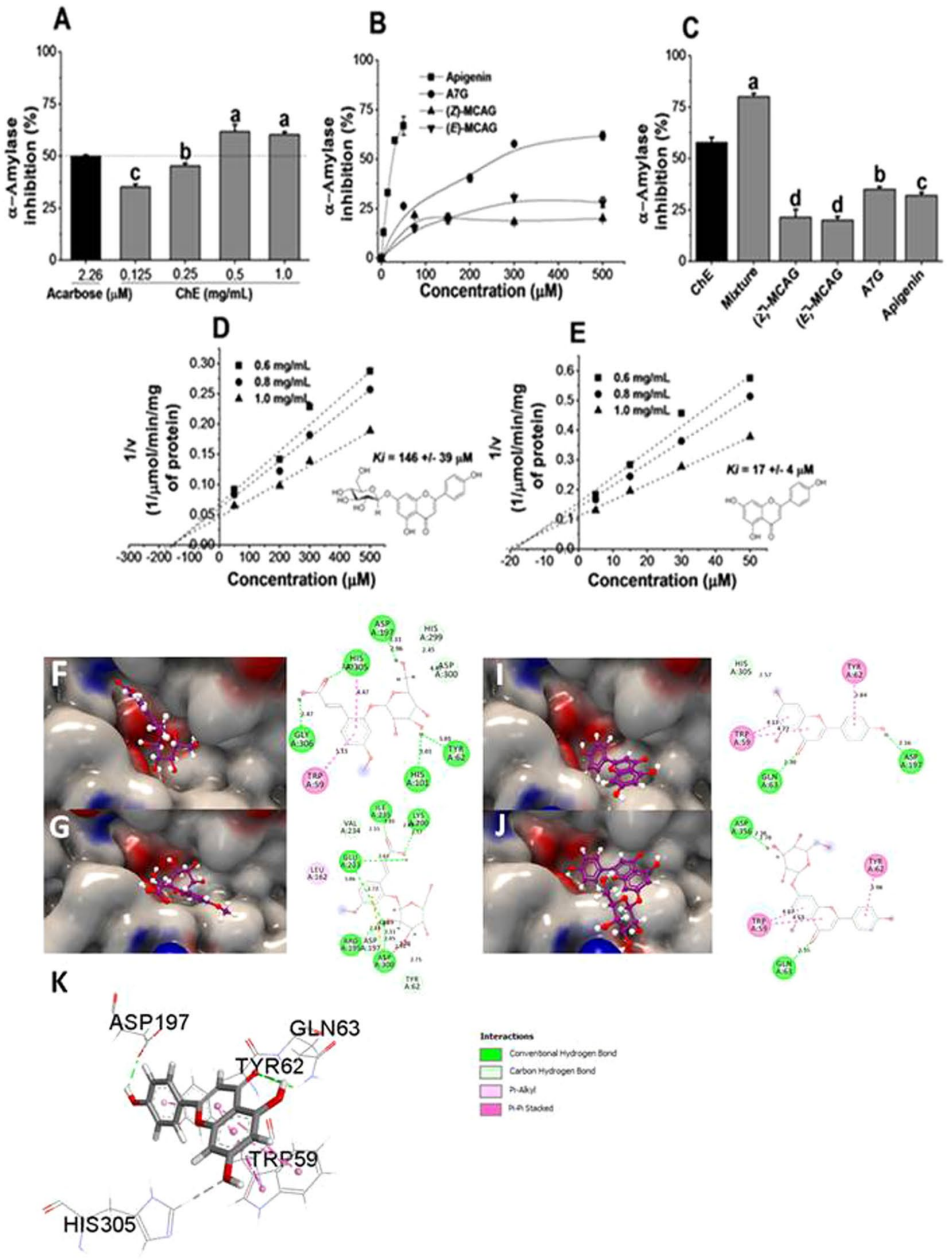
Inhibition of human α-amylase activity by ChE and component (poly)phenols. (**A**) Dose-dependent inhibition of ChE. IC_50_ for acarbose was obtained and used as positive control. (**B**) Dose-dependent inhibition by individual (poly)phenols. Inhibition is expressed as % compared to control incubations (no inhibitor; normalised to 100%). (**C**) Combined and individual inhibition of (poly)phenols at concentrations equivalent to those present in the ChE: (Z)-MCAG, 257 µM; (**E**)-MCAG, 200 µM; apigenin 7-O-glucoside (A7G), 148 µM; apigenin, 12 µM. (**D**,**E**) Dixon plot showing the kinetic analysis of apigenin and A7G against α-amylase. The intercept value represents –*Ki*. Data is expressed as mean ± SEM of three independent experiments with at least three technical replicates. When not visible, the error bars are smaller than the data point(s). (**F**–**J**) Charge surface representation (in blue and red) of α-amylase binding cavity with respective ligands displayed in stick format (in purple); (**F**) (*Z*)-MCAG, (**G**), (*E*)-MCAG, (**I**) apigenin and (**J**) A7G. Next to each 3D complex are schematic representations of the 2D interactions between each ligand and amino acid residues including the length of each interaction (in Å) as displayed by Discovery Studio software. (**K**) Close-up 3D view illustrating the H-bond and π-π stacking interactions of apigenin adjacent to amylase active site residues.

As we previously found that ChE could inhibit human α-amylase and rat maltase activity^3^ and further confirmed in this study (Fig. 4A), we tested the inhibitory activity of its individual component (poly)phenols. Apigenin and its precursor apigenin 7-*O*-glucoside exhibited the most potent inhibition against α-amylase as evidenced by the dose-dependent response (Fig. 4B). (Z)-MCAG and (*E*)-MCAG were also active, although they showed only modest inhibition with maximum values ~25 and ~20% respectively (*p* < 0.05) (Fig. 4B). When the four (poly)phenols were combined at concentrations equivalent to those quantified in the ChE preparation, apigenin 7-*O*-glucoside showed the highest contribution (*p* < 0.05) (Fig. 4C). Moreover, their combination resulted in a ~20% increase of the inhibitory activity when compared to ChE.

**Figure 5:**
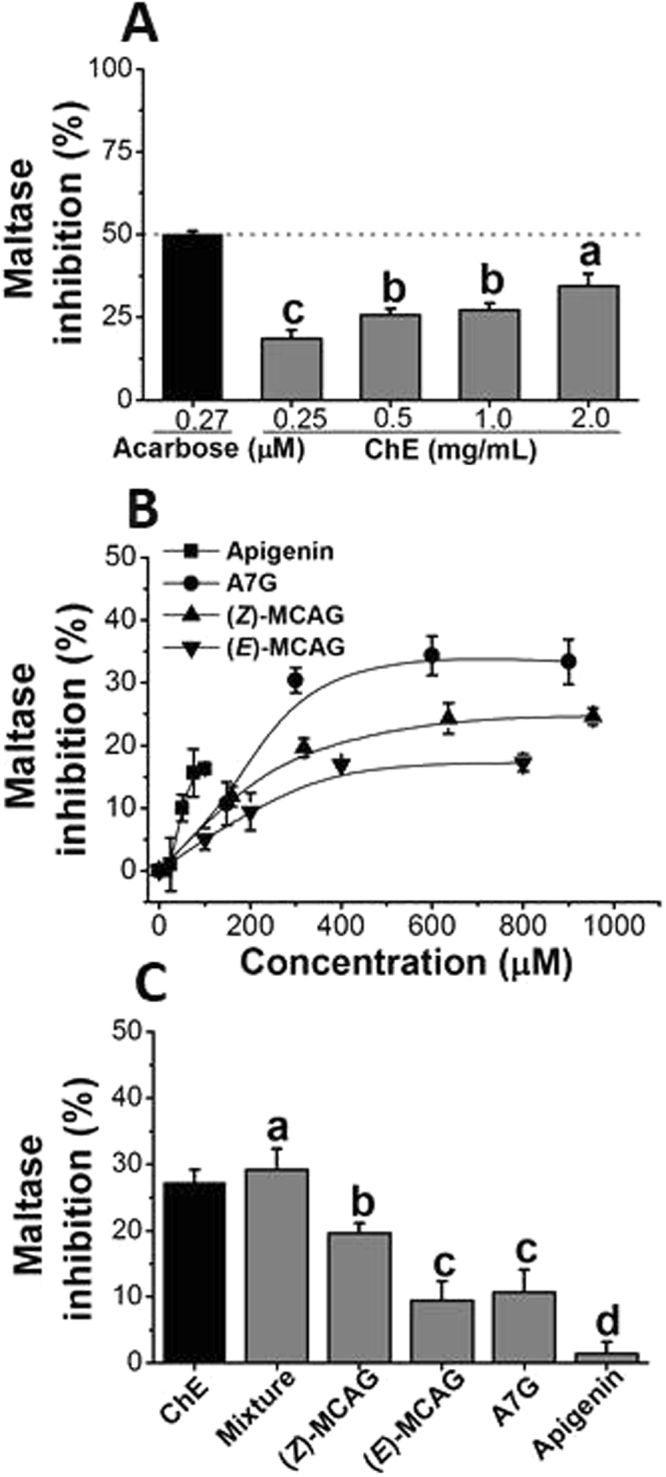
Inhibition of rat maltase activity by ChE and (poly)phenols. (**A**) Dose-dependent inhibition of ChE. IC_50_ for acarbose was obtained and used as positive control. (**B**) Dose-dependent inhibition of individual (poly)phenols. (**C**) Combined and individual inhibition effect of (poly)phenols at concentrations equivalent to those present in the ChE: (*Z*)-MCAG, 257 µM; (*E*)-MCAG, 200 µM; apigenin 7-*O*-glucoside (A7G), 148 µM; apigenin, 12 µM. Data is expressed as mean ± SEM of three independent experiments with at least three technical replicates.

While ChE weakly inhibited rat intestinal maltase, no inhibitory effect was observed on rat sucrase and isomaltase (Fig. 5A); even at the highest tested concentration only a modest ~33% (*p* < 0.05) inhibition was evident. When individual constituents were tested at concentrations equivalent to those present in ChE and higher, rat maltase was similarly only weakly inhibited by the individual (poly)phenols and apigenin 7-*O*-glucoside was the principal contributor (*p* < 0.05) (Fig. 5B,C). Nevertheless, a dose response was obtained for inhibition with ChE and the four individual compounds. Although apigenin was found to be the most potent inhibitor of the four constituent (poly)phenols, its limited solubility prohibited testing at concentrations >50 µM (Fig. 5B). None of the individual (poly)phenols or their combination achieved 50% inhibition. (*E)*-MCAG was resistant to digestive hydrolytic enzymes, indicating that 2-(*E*)-MCA would not be produced in significant amounts in the small intestine, and therefore 2-(*E*)-MCA was not assessed for inhibition of carbohydrate-digesting enzymes (supplementary S3).

#### Molecular docking studies

Molecular docking was performed to investigate the structural basis of the observed inhibition on human α-amylase by individual (poly)phenols. Based on the strong inhibition observed for apigenin, three important features were identified: (1) the formation of H-bonds between the OH group of the B ring and the catalytic residue Asp^197^, (b) the formation π-π stacking interactions with Trp^59^ and Tyr^62^ and (c) the stacking towards the binding subsites −1 and −2. As shown in supplementary S4, these observations were extended to other strong (poly)phenol inhibitors of human α-amylase (arbitrarily defined as molecules with IC_50_ <100 µM) including EGCG, quercetin and luteolin^18^. Apigenin 7-*O*-glucoside is able to form π-π stacking interactions with Trp^59^ and Tyr^62^, however it does not form the H-bond with the catalytic residue Asp^197^ resulting in a ~10 fold lower inhibition in agreement with the *in vitro* results (Fig. 4J).

(*Z*) and (*E*)-MCAG bind in a completely different pose (Fig. 4F,G) and are able to form H-bonds with the catalytic residues, but, unlike apigenin, the H-bonds are formed mainly with the OH groups of the glucose moiety and would be possibly weaker judging by the distance of H-bonds formed. In the case of (*Z*)-MCAG, an H-bond is formed between the 6-OH group of the glucose unit with Asp^197^ while (*E*)-MCAG presents a more complex interaction. In (*E*)-MCAG, 2 covalent bonds are formed between the carbon at position 2 and Asp^197^ (indicated as carbon-hydrogen bond in Fig. 4). Furthermore, H-bond interactions occur between the 6-OH group and Glu^233^ and Asp^300^ while an additional H-bond is formed between the Glu^233^ and the OH-present in the carboxylic group of the phenolic skeleton. These additional interactions would explain the slightly higher inhibition observed by the (*E*)-isomer (Fig. 4C) *in vitro*, and could perhaps account for its stability in the binding pocket given the lack of π-π stacking interactions.

### Inhibition of glucose, sucrose and fructose transport by ChE (poly)phenols

**Figure 6:**
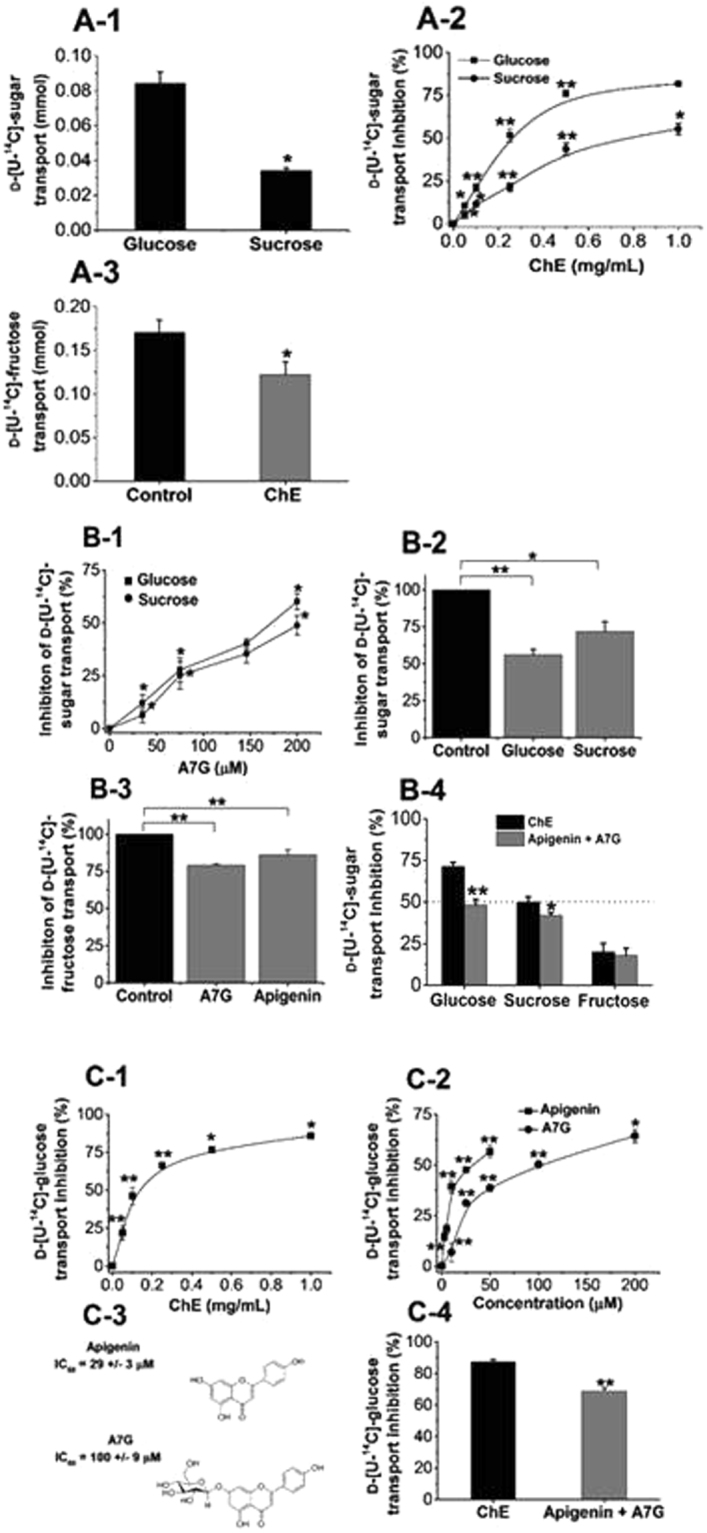
Monosaccharide transport across Caco-2/TC7 cells monolayers and inhibition by ChE and its major (poly)phenol constituents. (A-1) Transport D-[U-^14^C]-glucose and D-[U-^14^C]-sucrose in the absence of inhibitors. (A-2) Dose-dependent inhibition of D-[U-^14^C]-glucose and D-[U-^14^C]-sucrose transport by ChE. (A-3) Inhibition of D-[U-^14^C]-fructose by ChE at a single concentration (1 mg/mL). For D-[U-^14^C]-glucose and D-[U-^14^C]-sucrose transport the IC_50_ values were calculated as 0.24 ± 0.02 and 0.91 ± 0.06 mg/mL respectively. For D-[U-^14^C]-fructose, the maximum inhibition at the concentration tested was 28%. (B-1) Concentration-dependent inhibition of D-[U-^14^C]-glucose and D-[U-^14^C]-sucrose by apigenin 7-*O*-glucoside (A7G). (B-2) Inhibition of D-[U-^14^C]-glucose and D-[U-^14^C]-sucrose by apigenin at a single concentration (50 µM). (B-3) Inhibition of D-[U-^14^C]-fructose by A7G and apigenin at a single concentration; A7G (200 µM), apigenin (50 µM). (B-4) Inhibition of monosaccharide transport by ChE and combined inhibition of apigenin and A7G; apigenin, 12 µM; A7G, 148 µM. (C-1) Concentration-dependent inhibition of ChE under Na^+^-free conditions, IC_50_ = 0.138 ± 0.018 mg/mL. (C-2) Concentration-dependent inhibition of apigenin and A7G under Na^+^-free conditions. (C-3) Chemical structures of apigenin and A7G and their respective IC_50_ values under Na^+^-free conditions. (D) Combined inhibition of apigenin and A7G under Na^+^-free conditions; apigenin, 12 µM; A7G, 148 µM. Each data point represents the mean ± SEM of three independent experiments with six technical replicates. Results are expressed as % compared to the transported sugars in control incubations (no inhibitor; normalised to 100%). When not visible, the error bars are smaller than the data point. In Fig (A2), (B1), (C1 and C2) statistical difference is indicated against the incremental concentrations of each inhibitor. *p < 0.05, **p < 0.05.

Caco-2/TC7 cells transported 0.08 mmol of D-[U-^14^C]-glucose from the apical to the basolateral compartment across the cell monolayers in the presence of Na^+^, where the sugar transporter Na^+^/glucose co-transporter 1 (SGLT1) is active, in addition to the facilitated transporters GLUT2 and GLUT5 (Fig. 6A-1). D-[U-^14^C]-sucrose must first be hydrolysed by brush border sucrase and the resulting products, fructose and glucose, are transported into and across the cells. ChE dose-dependently inhibited D-[U-^14^C]-glucose, D-[U-^14^C]-sucrose (Fig. 6A-2) and D-[U-^14^C]-fructose transport (Fig. 6A-3).

In order to elucidate which (poly)phenols in ChE were responsible for the effect, each was tested separately on D-[U-^14^C]-monosaccharide transport across Caco-2/TC7 cell monolayers. Among individual (poly)phenols, apigenin 7-*O*-glucoside was a very effective inhibitor of D-[U-^14^C]-glucose and D-[U-^14^C]-sucrose transport, and IC_50_ values were obtained (Fig. 6B-1). Although an IC_50_ value could not be obtained for apigenin at the highest soluble concentration tested (Fig. 6B-2), it can be deduced that it is a more potent inhibitor based on the substantial 3-fold higher effect when compared with apigenin 7-*O*-glucoside at equivalent concentrations. Both (*Z*) and (*E*)-MCAG had no effect on D-[U-^14^C]-glucose, D-[U-^14^C]-sucrose or D-[U-^14^C]-fructose transport (data not shown). The combination of apigenin and apigenin 7-*O*-glucoside when tested at concentrations equivalent to those quantified in the extract, accounted for ~62, 80 and 89% of the total transport inhibition of D-[U-^14^C]-glucose, D-[U-^14^C]-sucrose and D-[U-^14^C]-fructose, respectively, exhibited by ChE (Fig. 6B-4).

To dissect the mechanism of inhibition of glucose transport, we conducted D-[U-^14^C]-glucose transport experiments under Na^+^-free conditions where only GLUT2, and not SGLT1, is active. An apparent increase (~50%) in the inhibitory activity was observed for ChE (*p* < 0.05), apigenin 7-*O*-glucoside and apigenin as evidenced by the decrease in the inhibition constants, suggesting that the main inhibitory effect is on GLUT2 (Fig. 6C-1 and 2). When tested in combination under Na^+^-free conditions, apigenin 7-*O*-glucoside and apigenin (Fig. 6C-3) were responsible for ~80% of the inhibitory activity observed by the whole ChE (*p* < 0.05) (Fig. 6C-4).

**Figure 7:**
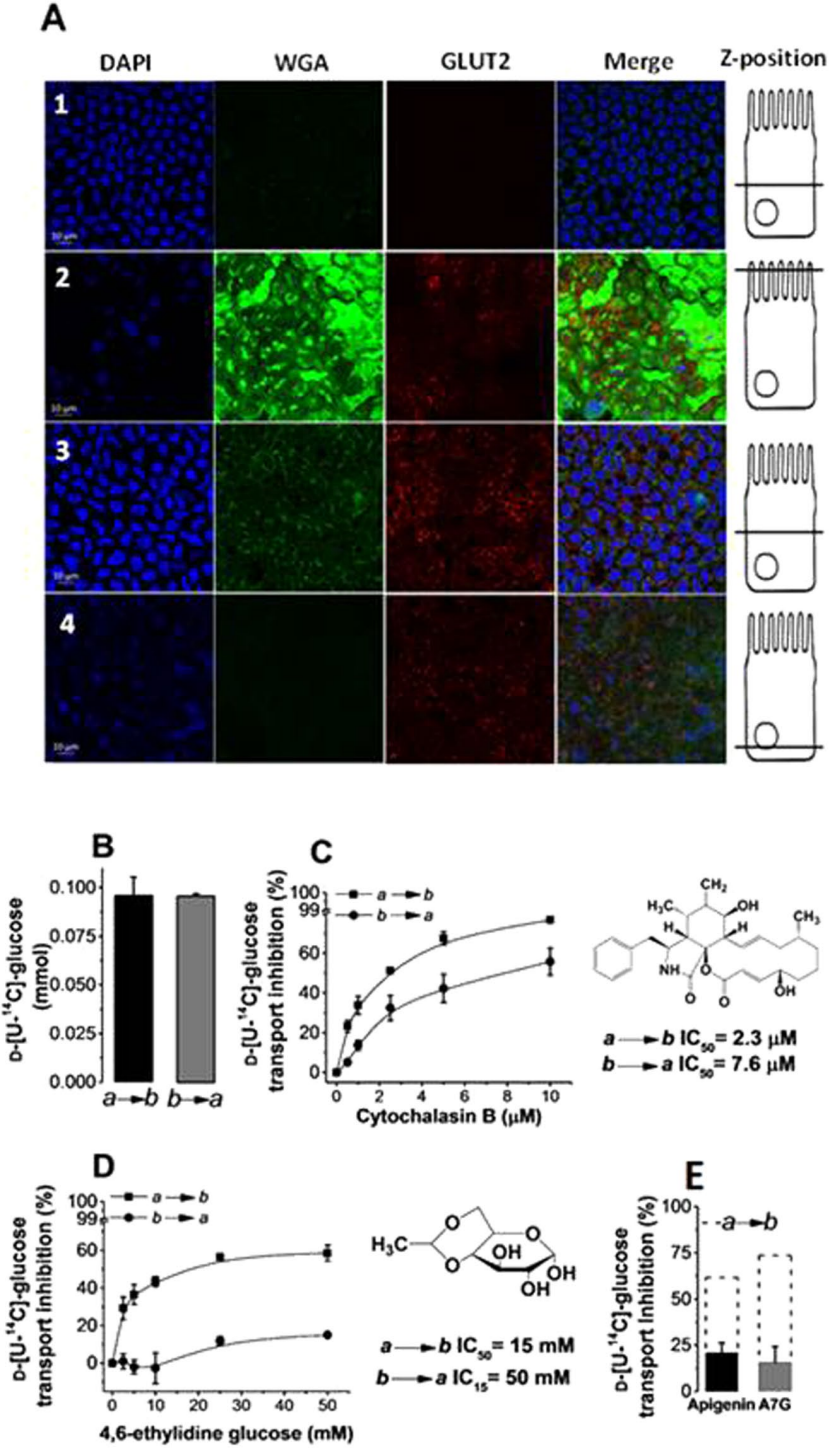
Characterisation of the binding site of apigenin and apigenin 7-O-glucoside (A7G) on GLUT2. Transport experiments were conducted under Na^+^-free conditions. (A) Immunofluorescence detection of GLUT2 in differentiated Caco-2/TC7 cell monolayers. Row 1 shows control cell layer incubated with 4–6-diamidino-2-phenylindole (DAPI) and Cy3-conjugated donkey anti-rabbit secondary antibody only. Cells in rows 2–4 were incubated with DAPI, membrane marker wheat germ agglutinin (WGA), GLUT2 primary antibody and Cy3-conjugated donkey anti-rabbit secondary antibody. GLUT2 is shown in red, appearing orange when co-localising with WGA shown in green. Nuclei are stained with DAPI (blue). Scale bars (10 µm) are shown in the lower left corner of DAPI images. Scale bar applies to all imagesin the row. Images are representative examples of three independent immunostaining experiments. (**B**) Asymmetric D-[U-^14^C]-glucose transport without inhibitor. (**C**) Asymmetric D-[U-^14^C]-glucose transport in the presence of cytochalasin B and inhibition constants. (**D**) Asymmetric D-[U-^14^C]-glucose transport in the presence of 4,6-ethylidene glucose and inhibition constants. (**E**) Inhibition of asymmetric D-[U-^14^C]-glucose transport by apigenin and A7G. Dotted line represents the inhibition values obtained at the maximum concentration tested when D-[U-^14^C]-glucose transport was assessed from apical (*a*) to basolateral (*b*) (data replotted from Fig. 6C-2). Data is expressed as mean ± SEM of three independent experiments with at least three technical replicates. In panel (**B,C**) results are expressed as % compared to the transported glucose in control incubations (no inhibitor; normalised to 100%). When not visible, the error bars are smaller than the data point. In panel (**B**) and (**C**), statistical difference is indicated against the incremental concentration of cytochalasin B and 4,6-ethylidene glucose respectively. **p < 0.05.

Since the main inhibition we found was on GLUT2, we investigated the binding affinities of apigenin and apigenin 7-*O*-glucoside. Immunostaining of GLUT2 showed that GLUT2 protein was present on both apical and basolateral sides of differentiated Caco-2/TC7 cells (Fig. 7A). This was also confirmed by functional assays where the rate of D-[U-^14^C]-glucose transport under Na^+^-free conditions was the same from apical to basolateral compartments and *vice versa* (Fig. 7B). Inhibition constants for the exofacial glucose transporter inhibitor 4,6-*O*-ethylidene glucose and the endofacial glucose transporter inhibitor cytochalasin B on basolateral to apical D-[U-^14^C]-glucose transport were compared to those for apigenin and apigenin 7-*O*-glucoside (Fig. 7C–E). The results suggest that the two (poly)phenols bind at the exofacial side of the protein, since a 3-fold lower inhibition was observed when the D-[U-^14^C]-glucose transport was assessed from basolateral to apical side, which was also observed for 4,6-*O*-ethylidene glucose. This comparison is made assuming that the position of GLUT2 is the same when facing the apical and the intracellular space, which seems to hold based on the rate of D-[U-^14^C]-glucose transport across the monolayers in both directions.

### Caco-2/TC7 cells do not convert apigenin 7-*O*-glucoside to apigenin

Glycosylated (poly)phenols can be hydrolysed by luminal β-glucosidase enzymes, such as lactase phlorizin hydrolase^19,20^, present in the brush border of the enterocytes, resulting in the release of the aglycone. Therefore, we explored the possibility whether the inhibition observed by apigenin-7-*O*-glucoside was due to the presence of apigenin after enzymic hydrolysis. Caco-2/TC7 cells were incubated with 150 µM apigenin 7-*O*-glucoside for 25 and 60 min. *In vitro* conversion of apigenin 7-*O*-glucoside to apigenin in Caco2/TC7 cells was negligible (Supplementary S5) confirming that apigenin 7-*O*-glucoside can interact directly with GLUT2 transporter protein.

## Discussion

Repeated high postprandial glucose spikes and fructose related metabolic burden have been proposed as independent risk factors for developing insulin resistance and progression to type 2 diabetes^21–24^. Delaying the absorption of sugars in the intestine is considered a clinically relevant approach for reducing type 2 diabetes risk^22,23^. The present study was designed to identify the (poly)phenols involved in the inhibition of carbohydrate digestion and absorption previously demonstrated by ChE^3^ and further explore their mechanism of action.

In the work presented here we have provided conclusive evidence of the presence of (*Z*) and (*E*)−2-β-D-glucopyranosyloxy-4-methoxycinnamic acids in ChE, in agreement with the tentative identification of Weber *et al*.^17^. These compounds were previously misidentified as ferulic acid hexosides and ‘dimer’ forms by several authors^11,14,15,25^. When they were individually tested, they showed modest inhibitory activity on human α-amylase; however, when assessed in combination with apigenin and apigenin 7-*O*-glucoside, it was evident that they significantly contribute to the overall bioactivity of ChE.

*In vitro* and *in silico* studies revealed that apigenin and apigenin 7-*O*-glucoside are primarily responsible for the inhibition of human α-amylase by ChE, and this arises in part from their ability to occupy the binding pocket and stack between subsite “−1 and −2” through π-π interactions. The side chain residue of Asp^197^ is believed to act as nucleophile during the catalytic reaction of α-amylase and its substitution dramatically decreased (10^6^-fold) its catalytic activity^26^. Unlike apigenin 7-*O*-glucoside, apigenin showed additional interaction with Asp^197^ through an H-bond and this was reflected in a ~10-fold increase in its inhibitory activity. Thus, H-bonds between OH groups of the B ring and catalytic residues, and π-π stacking interactions appear to be requirements for an optimal inhibition of human α-amylase by (poly)phenols acting *via* a competitive mechanism. Indeed, in a previous study, Lo Piparo *et al*.^27^ observed similar features; (poly)phenols showing IC_50 _<100 µM were anchored in the active site through π-π interactions with Trp^59^, and formed H-bonds with residues Asp^197^ and Glu^233^ through OH groups in the A and B rings. This mechanism is further supported by X-ray structural analyses of the (poly)phenols myricetin and montbretin A with human pancreatic α-amylase^28,29^.

The final step of starch digestion is catalysed by α-glucosidases, maltase-glucoamylase and sucrase-isomaltase. Their combined catalytic activities hydrolyse linear and branched malto-oligosaccharides after the action of pancreatic α-amylase^30^ while sucrase-isomaltase additionally hydrolyses sucrose, producing glucose and fructose. Inhibition of α-glucosidases is complementary in strategies aiming at blunting blood sugar spikes stemming from consumption of readily absorbable sugars which increase the prospect of type 2 diabetes.^31,32^ Our results indicate only a modest inhibition of maltase-glucoamylase by the four (poly)phenols, even when tested in combination, consistent with the inhibition observed for ChE. In contrast, Kato *et al*.^5^ found a stronger inhibition of ChE against maltase-glucoamylase (IC_50_ = 2.6 mg/mL) and sucrase-isomaltase (IC_50_ = 0.9 mg/mL) using brush border membranes prepared from rat small intestine. The authors suggested that this effect was driven by the presence of esculetin (maltase IC_50_ = 534 μM; sucrase IC_50_ = 72 μM) and quercetin aglycone (maltase IC_50_ = 216 μM; sucrase IC_50_ = 71 μM). However, the phytochemical composition and content of the plant material used in the study was not reported. Data from other studies on chamomile composition indicate that these compounds, if present at all, are unlikely to be effective since they are several orders of magnitude below the concentrations necessary for inhibition^11,33,34^.

At low concentrations, the uptake of glucose and fructose relies on SGLT1 and the facilitated transporter GLUT5, respectively. However, after consumption of a meal high in sugars, glucose concentration may reach 50–300 mM in the intestinal lumen prompting the facilitated GLUT2 transporter to translocate to the apical membrane. GLUT2 accounts for ~75 and ~60% of total glucose and fructose uptake respectively.^35,36^ Our data show that apigenin and apigenin 7-*O*-glucoside are potent and selective inhibitors of GLUT2 as these compounds account for up to ~80% of transport inhibition under Na^+^-depleted conditions. They bind at the exofacial site of the protein as demonstrated by comparing binding affinities with specific inhibitors of the intra- and extracellular cavities of the transport channel. In contrast, apigenin and apigenin 7-*O*-glucoside showed a modest inhibition of fructose transport and only a small fraction of the transport of D-[U-^14^C]-fructose was inhibited following hydrolysis of D-[U-^14^C]-sucrose by brush-border α-glucosidase. However, a higher inhibition of fructose transport in a physiological context can be expected since the apical expression of GLUT5 in the Caco-2 clone is comparable to the one noted in an adult small intestine^37^ and Caco-2/TC7 cell line presents a 2-fold increase in apical GLUT5 compared to Caco-2 cells^3^.

Like other (poly)phenols, apigenin and apigenin 7-*O*-glucoside are bioavailable in humans but are rapidly metabolised and excreted, while they are subject to extensive colonic catabolism^38,39^. Currently, there are no pharmacokinetic data on (*Z*)- or (*E*)-MCAG but it is anticipated that they can reach the colon and are subject to catabolic reactions as observed for other hydroxycinnamic acids^40^, especially given that (*E*)-MCAG was not a substrate for rat intestinal enzymes. Based on information from other related compounds^41,42,^ no harmful effects are predicted after dietary exposure of these (poly)phenols through consumption.

Several papers have reported that apigenin in various herbal and plant extracts can inhibit α-glucosidase activity. For example, apigenin-7-O-glucoside inhibited rat intestinal α-glucosidase activity, but the study used *p*-nitrophenyl-glucopyranoside, rather than the physiological substrate (maltose)^43^. Many studies have used yeast as a source of α-glucosidase activity, which has no relevance to the mammalian enzymes^44^. Hence apigenin did not inhibit yeast α-glucosidase action on the non-physiological substrate *p*-nitrophenyl-glucopyranoside in one report^45^, but did inhibit in other reports with an IC_50_ value of 22 μM in^46^ and of 25.5 μg/ml in^47^. In the latter report, apigenin-7-*O*-glucoside also inhibited with an IC_50_ value of 29.7 μg/ml^47^. Apigenin lowered GLUT1 expression in a nude mouse model of laryngeal carcinoma^48^ and in prostate cancer cells^49^.

Based on our data, we propose that ChE, a natural source that combines (*Z*) and (*E*)-MCAG, apigenin and apigenin 7-*O*-glucoside, could pose a good alternative to modulate postprandial sugar excursions. Unlike the antidiabetic drug acarbose, the combination of these (poly)phenols can mildly attenuate carbohydrate digestion and sugar absorption, avoiding the gastrointestinal side effects of acarbose. Furthermore, apigenin and apigenin 7-*O*-glucoside could be employed either alone or in combination with other inhibitors for an enhanced formulation, for example in a population that has already developed a pathophysiological phenotype.

## Materials and Methods

### Preparation of ChE

The ChE used in this study and in enzymic *in vitro* assays has been previously fully described.^3^

### Isolation of hydroxycinnamic acid derivatives by semi-preparative column chromatography

Two hydroxycinnamic acid derivatives detected based on previously published multiple reaction monitoring pairs by LC-MS were separated and isolated using an ÄKTA Purifier System (GE Healthcare Life Sciences, UK) equipped with an Gemini C6 phenyl column (250 × 10 mm I.D., 5 μm; Phenomenex, Cheshire, UK). The ChE extract (150 mg/mL) was loaded manually (1 mL) using a syringe through a sample loop of 1 mL and eluted using water containing 0.1% TFA (solvent A) and methanol (Solvent B) at a flow rate of 2.3 mL/min as follows: 0–12.8 min linear gradient to 24% B; 12.8–25.6 min isocratic at 24% B; 25.6–111 min linear gradient to 100% B; 111–123.8 min isocratic at 100%B, 123.8–125 min linear gradient to 5% B: 125–150.6 min isocratic at 5%B. The elution was followed at 320 nm and fractions containing the separated compounds (supplementary S1) were collected and analysed for purity by LC-MS (supplementary S6). Multiple semi-preparative separation cycles were undertaken and the fractions for each peak combined, freeze-dried, and stored at −20 °C.

### Chemical characterisation of hydroxycinnamic acid derivatives

#### HPLC/QTOFMS

Analysis of the purified hydroxycinnamic acid derivatives was carried out using an Agilent 1200 series HPLC system coupled to an Agilent 6530 Q/TOF mass spectrometer (Agilent Technologies, USA) equipped with a dual spray ESI source operating in negative mode. Followed by an isocratic separation with reversed phase HPLC, elemental formulae of compounds were determined with the aid of MassHunter software based on high-resolution (>20,000 FWHM) accurate mass (<5 ppm) data completed with evaluation of isotope abundance matching and isotope spacing. Data was obtained from full-scan TOF-only peak spectra acquired in the mass range of *m/z* 50–1100. Next, manually selected ion peaks of the TOF-only spectra were subjected to Q/TOFMS analysis and elemental formulae of fragments were determined based on accurate mass (<20 ppm) data obtained from product ion peak spectra.

#### Acid hydrolysis

An aliquot of the purified compound 2 (Fig. 1 and supplementary S1) was dissolved in HCl (2 M) to a concentration of 500 µM and subjected to hydrolysis for 2 h at 60 °C. The reaction mixture was concentrated to dryness under vacuum, reconstituted in water (1 mL) and analysed by HPLC-DAD on an Agilent 1200 series system (Agilent Technologies, USA) equipped with a Kinetex C18 analytical column (150 × 2.1 mm I.D., 2.6 μm; Phenomenex, Cheshire, UK) maintained at 35 °C. 10 µL was injected and separated using a 41 min gradient of premixed 5% acetonitrile in water (5:95, v/v) (A) and premixed 5% water in acetonitrile (5:95, v/v) (B), both modified with 0.1% formic acid at 0.25 mL/min. The gradient started at 0% solvent B and increased to 10% (5 min), 25% (10 min), 35% (20 min), 50% (25 min), and was held at a plateau up to 30 min. The gradient was increased 100% at 30.5 min and returned to 0% solvent B over 5.5 min before initial starting conditions were resumed for a 6 min column re-equilibration. Online detection was carried out at 320 nm.

A second aliquot was subjected to hydrolysis, concentrated to dryness and derivatised with 1-phenyl-3-methyl-5-pyrazolone (PMP) as described elsewhere^50,51^. The derivatised sugar was analysed on HPLC-DAD equipped with a C18 analytical column (250 × 4.6 mm I.D., 5 µm, Phenomenex, Cheshire, UK) and UV detection at 250 nm. 10 µL of sample was eluted with 0.1 mM ammonium acetate buffer (pH 5.5)–acetonitrile (78:22, v/v) at 1 mL/min. The identification of the glycosyl component was done by comparing the retention time with those of authentic standards (D-mannose, D-glucose, D-galactose and D-xylose).

#### NMR

Purified samples were dissolved in d_6_ DMSO and transferred to 5 mm OD NMR tubes. All NMR data were recorded on a Bruker Avance 500 spectrometer (^1^H frequency 500.573 MHz, ^13^C frequency 125.878 MHz) temperature controlled at 299.3 K. Chemical shifts are expressed in ppm and are reported with reference to the residual solvent peak. Multiplicities are reported with coupling constants and are given to the nearest 0.1 Hz. Where needed, two-dimensional correlation spectroscopy (2D-COSY), heteronuclear single quantum coherence spectroscopy (HSQC) and heteronuclear multiple bond correlation spectroscopy (HMBC) and nuclear Overhauser effect spectroscopy (NOESY) was performed.

(*Z*)-2-D-Glucopyranosyloxy-4-methoxycinnamic acid present as a mixture of E and Z in a 1:3 ratio, E peaks eliminated and Z peaks picked at: ^1^H NMR (501 MHz, DMSO-d6) δ 7.70 (d, *J* = 8.5 Hz, 1 H, H3), 7.19 (d, *J* = 12.5 Hz, 1 H, *cis*-alkene, H2), 6.74 (d, *J* = 2.5 Hz, 1 H, H5), 6.56 (dd, *J* = 8.5, 2.5 Hz, 1 H, H4), 5.78 (d, *J* = 12.5 Hz, 1 H, *cis*-alkene, H1), 4.85 (d, *J* = 7.5 Hz, 1 H, anomeric proton, H8), 3.77 (s, 3 H, H6), 3.72 (broad d, *J* = 11.7 Hz, 1 H, H13), 3.47 (broad dd, *J* = 11.7, 6.0 Hz, 1 H, H13′), 3.38 (ddd, *J* = 10.0, 6.2, 1.6 Hz, 1 H, H12), 3.34–3.29 (m, 2 H, H9 & H10)*, 3.16 (apparent t, *J* = 8.8 Hz, 1 H, H11) (. ^13^C NMR (126 MHz, DMSO-d6) δ 167.7, 161.0, 160.7, 156.7, 136.32, 131.1, 117.53, 116.73, 101.11, 100.5, 77.24, 76.86, 73.27, 69.81, 60.72, 55.33. *Overlayed by strong signal from water. ESI-MS: *m/z* 355.1035 [M-H]^-^. The ^1^H assignments in the chemical structure are shown in supplementary S7-A.

Analysis of pure (*E*)−2-β-D-Glucopyranosyloxy-4-methoxycinnamic acid: ^1^H NMR (501 MHz, DMSO-d6) δ 12.00 (s, 1 H, H7), 7.82 (d, *J* = 16.2 Hz, 1 H, *trans*-alkene, H2), 7.62 (d, *J* = 8.7 Hz, 1 H, H3), 6.76 (d, *J* = 2.4 Hz, 1 H, H5), 6.62 (dd, *J* = 8.6, 2.4 Hz, 1 H, H4), 6.39 (d, *J* = 16.2 Hz, 1 H, *trans*-alkene, H1), 5.16 (broad s, 1 H (OH)), 4.98 (d, *J* = 7.5 Hz, 1 H, anomeric proton, H8), 4.89 (broad s, 2 H (OH)), 4.58 (broad s, 1 H, (OH)), 3.78 (s, 3 H, H6), 3.72 (broad d, *J* = 11.7 Hz, 1 H, H13), 3.47 (broad dd, *J* = 11.7, 6.0 Hz, 1 H, H13′), 3.38 (ddd, *J* = 10.0, 6.2, 1.6 Hz, 1 H, H12), 3.34–3.29 (m, 2 H, H9 & H10)*, 3.16 (apparent t, *J* = 8.8 Hz, 1 H, H11). ^13^C NMR (126 MHz, DMSO-d6) δ 168.08, 162.08, 157.07, 138.76, 129.32, 116.73, 116.05, 108.03, 101.11, 100.02, 77.24, 76.86, 73.27, 69.81, 60.72, 55.33. *Overlayed by strong signal from water. ESI-MS: *m/z* 355.1035 [M-H]^-^. The ^1^H assignments in the chemical structure are shown in supplementary S7-B. NOESY spectrum and key enhancements are shown in supplementary S8 and Fig. 3, respectively.

#### Synthesis of (E)−2-hydroxy-4-methoxycinnamic acid

7-Methoxy coumarin (0.44 g, 2.5 mmol) was dissolved in an aqueous solution of 0.5 M NaOH (20 mL) and stirred for 4 h at room temperature. Aliquots were taken and showed full conversion to the *trans* carboxylate by NMR in D_2_O. The solution was then acidified with 1 M aqueous HCl and extracted with dichloromethane (3 × 10 mL). The solvent was then evaporated and eluted with ethyl acetate on a flash silica column. This yielded the (*E*)−2-hydroxy-4-methoxycinnamic acid (*E-*MCA) (32 mg, 0.165 mmol, 6.6% yield). The low yield was due to the ring closing to reform the coumarin in acidic conditions. ^1^H NMR (500 MHz, acetone) δ 9.25–9.21 (broad s, 1 H, H7), 7.95 (d, *J* = 16.1 Hz, 1 H, H2), 7.54 (d, *J* = 8.5 Hz, 1 H, H3), 6.40 (d, *J* = 2.2 Hz, 1 H, H5), 6.37 (dd, J = 2.2, 8.5 Hz, 1 H, H4) 6.33 (d, J = 16.1 Hz, 1 H, H1), 3.66 (s, 3 H, H6). The ^1^H assignments in the chemical structure are shown in supplementary S7-C.

The synthesised compound was analysed by HPLC-DAD as described in section 2.3. ESI-MS: *m/z* 193.06 [M-H]^-^ **(**supplementary S9**)**.

### Enzyme assays

The human α-amylase and rat α-glucosidase inhibition assays were performed under optimised conditions as previously reported^3,18,52^. The ChE stock solution was dissolved in DMSO while the individual (poly)phenols were prepared in a water-EtOH solution (50:50, v/v). The maximum concentration of DMSO and EtOH in the assay was 2 and 1% respectively, which did not affect the activity of the enzyme as assessed in control incubations.

### Molecular Modelling

We investigated the interactions of (poly)phenols with human α-amylase using the three-dimensional crystal structure 1MFV^53^. The α-amylase structure was prepared for docking by removing from the crystallographic structure water molecules and co-crystallised ligands. No constraints were introduced and the default parameters of the dock module of the CLC Drug Discovery 3.0.1 (CLC Bio-Qiagen) were used. Validation of the docking parameters was performed by docking the co-crystal modified structure of acarbose into the active site. A root mean square deviation of 1.36 Å was obtained between the original and the docked conformations (supplementary S10). The number of docking iterations for evaluation of the preferred binding pose was set at 500. Ten best docked poses per docking calculation were analysed based on their docking score and the top ranked pose for each ligand was selected for further analysis. Ligand-receptor interactions were visualised with CLC workbench and Discovery Studio (BIOVIA, Client version 2016).

### Transport assay of monosaccharides in Caco-2/TC7 cells

Caco-2/TC7 cells were cultured and transport experiments performed as fully described before^3^.

### Immunofluorescence staining

Caco-2/TC7 cells were seeded at 7 × 10^4^ cells/well on 12 well Millicell cell culture inserts (PET 0.4 mm pore size, Millipore) and maintained for 21–23 d at 37 °C (10% CO_2_). For staining, cells were fixed with 4% *para*-formaldehyde in PBS and incubated with wheat germ agglutinin (WGA, 1 mg/mL) for 10 min at 37 °C. Following permeabilisation with 0.1% Triton-X100 for 20 min at room temperature, cells were incubated with GLUT2 antibody (ab95256, Abcam, Cambridge, UK) at a dilution of 1:50 for 1 h, or without primary antibody for controls. After washing with PBS, cells were further incubated with Cy3-conjugated donkey anti-rabbit IgG at a 1:300 dilution. Cell layers were stained with 2 μg/mL 4′−6-diamidino-2-phenylindole (DAPI) and mounted on microscope slides with ProLong Gold antifade reagent mounting medium (Carlsbad, USA) and imaged using a Zeiss LSM700 confocal microscope with a Plan-Apochromat 63 × oil immersion objective.

### Estimation of conversion of apigenin 7-*O*-glucoside to apigenin by differentiated Caco-2/TC7 cells

Cells were seeded at 2.4 × 10^5^ cells/well on 6-well plates (Corning, Appleton Woods, UK) and cultured for 21–23 d at 37 °C as described above. Cells were exposed to 150 μM of apigenin 7-*O*-glucoside for 25 and 60 min. After the treatment the medium was collected and analysed by HPLC as detailed above.

### Statistical analysis

Statistical analysis was performed by one-way ANOVA using the Number Cruncher Statistical System version 6.0 software (NCSS, LLC). Significant differences were assessed with Tukey-Kramer multiple comparison test (p < 0.05). Independent samples *t-*test was used to compare means of the contribution of apigenin 7-*O*-glucoside and apigenin to the total inhibition of sugar transport. Data are expressed as the mean ± standard error of mean (SEM).

### Data availability statement

Data available on the Open Science Framework (ID: osf.io./fs32u).

## Electronic supplementary material

Supplementary figures
