# Supplementary material for: Acute metabolic actions of the major polyphenols in chamomile: an in vitro mechanistic study on their potential to attenuate postprandial hyperglycaemia
Source: Sci Rep. 2018 Apr 3;8:5471. doi: 10.1038/s41598-018-23736-1 (PMC5882934; doi:10.1038/s41598-018-23736-1)

**Acute metabolic actions of the major polyphenols in chamomile: an *in vitro* mechanistic study on their potential to attenuate postprandial hyperglycaemia.**

Jose A Villa-Rodriguez^1^, Asimina Kerimi^1^, Laszlo Abranko^1,4^, Sarka Tumova^1^, Lauren Ford^2,3^, Richard S. Blackburn^3^, Christopher Rayner^2^, Gary Williamson^1*^.

**Supplementary S1.** Semi-preparative purification (*Z*)-MCAG (compound A) (*E*)-MCAG (compound B) in maltodextrin-free ChE.

**Supplementary S2.** MS analysis of compound 2 after acid hydrolysis. Acid hydrolysis was carried out as described in section 2.5.

**Supplementary S3. Enzymatic hydrolysis of Kaempferol 3-O-glucoside (positive control) and (E)-MCAG.** (A) Kaempferol 3-*O*-glucoside standard (300 µM). (B) Kaempferol standard (100 uM). (C) Kaempferol 3-*O*-glucoside hydrolysed with hesperidinase. (D) Kaempferol 3-*O*-glucoside hydrolysed with acetone rat intestinal extract. (E) Kaempferol 3-*O*-glucoside hydrolysed with pancreatin. (F) (E)-MCAG (500 µM) hydrolysed with hesperidinase. (G) (E)-MCAG (500 µM) hydrolysed hydrolysed with acetone rat intestinal extract. The enzymatic hydrolysis were conducted with 50 mg/mL of the respective enzyme for 3 h at 37 °C.

**Supplementary S4. The binding pose and chemical interactions of EGCG, quercetin and luteolin (in descending order) with human salivary α-amylase.** Next to each 3D complex are schematic representations of the 2D interactions between each ligand and amylase amino acid residue including the length of each interaction (in Å) as displayed by Discovery Studio software.

**Supplementary S5. *In vitro* conversion of apigenin 7-*O*-glucoside (A7G) to apigenin by Caco-2/TC7 cells.** (A) A7G standard (150 µM). (B) Incubation of A7G (150 µM) with Caco-2/TC7 cells/TC7 cells for 25 min. (C) Incubation of A7G with Caco-2/TC7 for 60 min (150 µM).

**Supplementary S6. Total ion chromatogram and mass spectra of isolated compound 1 (A) and compound 2 (B).**

**Supplementary S7. ^1^H proton assignment.** (A) Z-MCAG. (B) E-MCAG. (C). MCA.

**Supplementary S8. NOESY spectrum of (E)-2-β-D-glucopyranosyloxy-4-methoxycinnamic acid.**

**Supplementary S9. HPLC-MS analysis of chemically synthesised *E*-MCA.** Sample was analysed under the chromatographic conditions described in section 2.3.

**Supplementary S10. Validation of the docking program using the crystal structure of the human salivary α-amylase (1MFV) complexed with a modified acarbose ligand.** The figure shows the superimposition of the native conformation (in grey) with the docked one (in turquoise) in the active site. The RSMD of was within the acceptance deviation with values of 1.36. The small spheres in blue (calcium) and green (chlorine) represent cofactors.

**Supplementary S1.**

**Supplementary S2.**

***m/z* ^(-)^ Peak eluted at 13.4 min**

***m/z*^(+)^ Peak eluted at 13.4 min Peak eluted at 15.3**

**^^**

**Supplementary S3**

**Supplementary S4.**

**
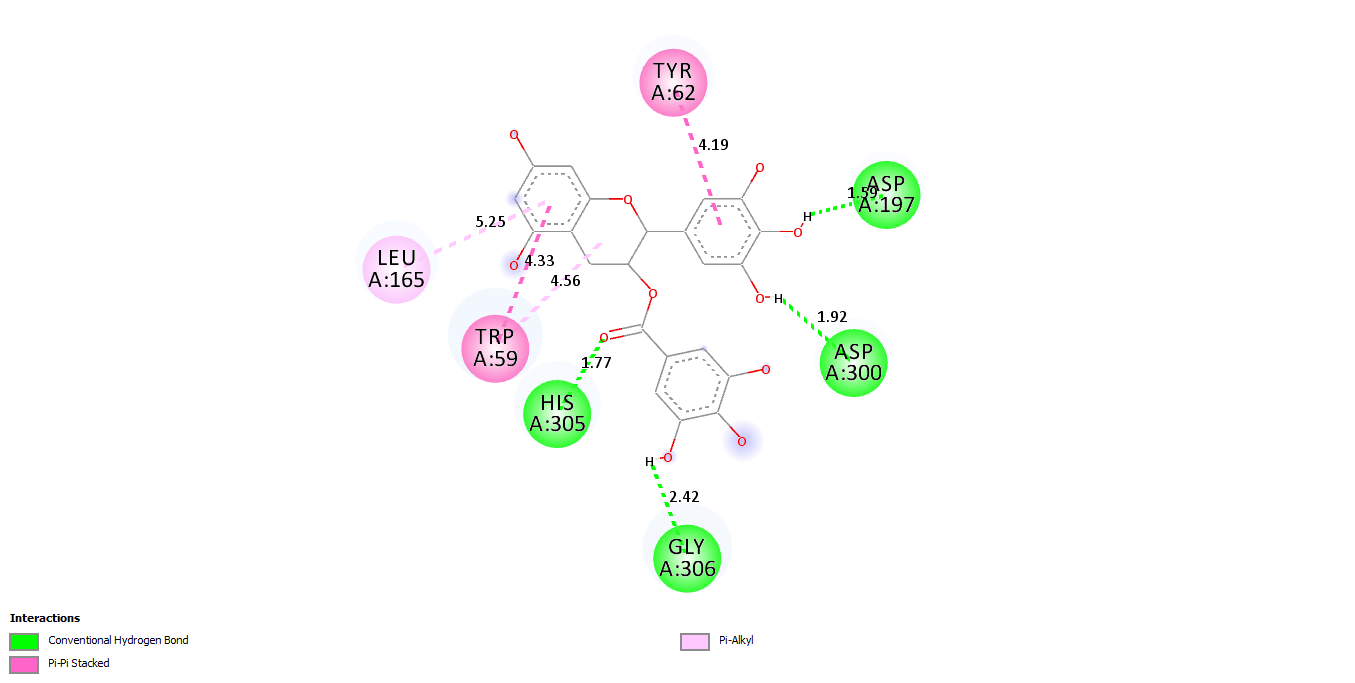

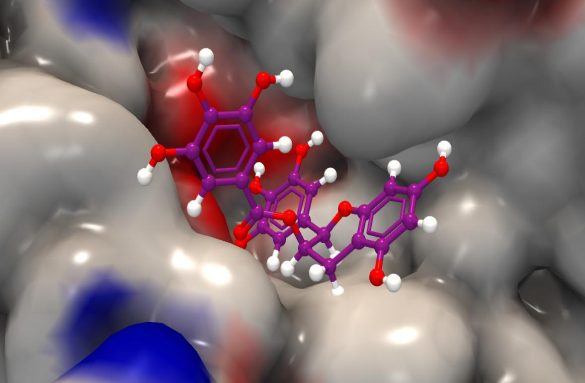

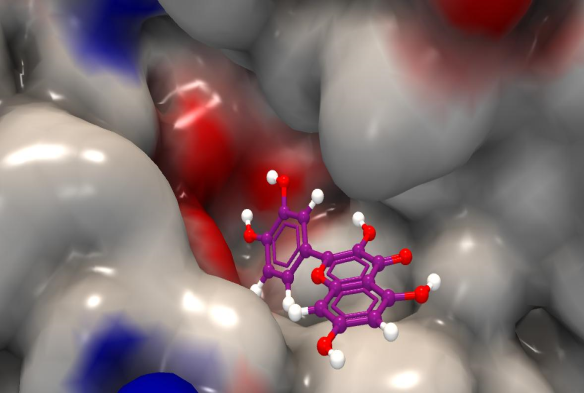
**

**
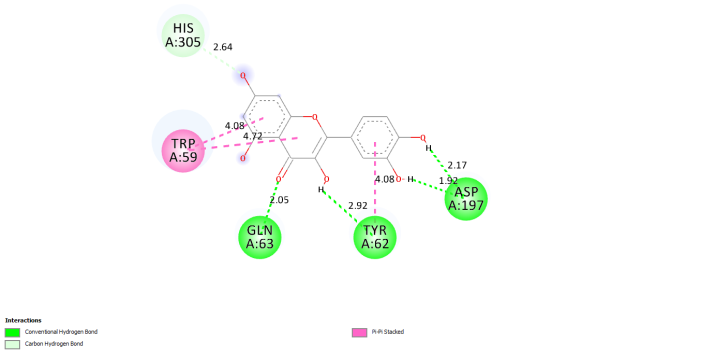
**

**
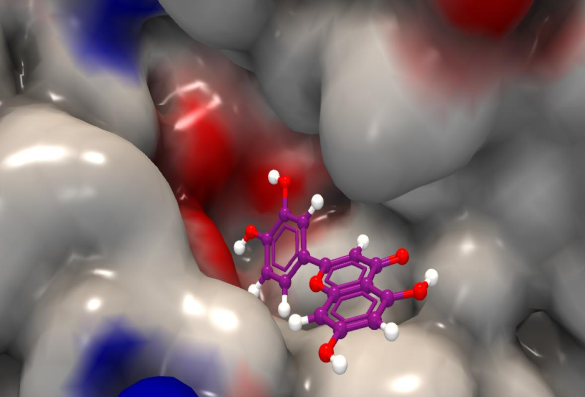
**

**
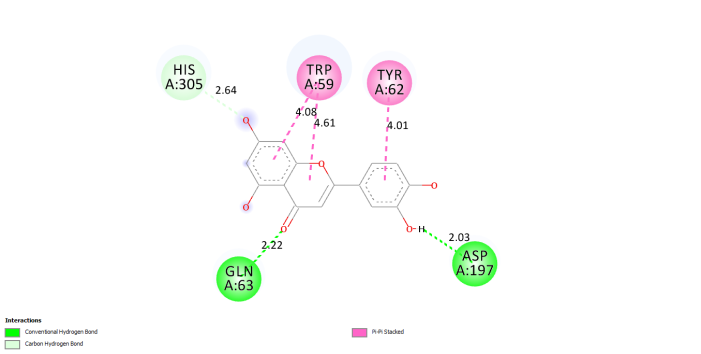
**

**Supplementary S5.**

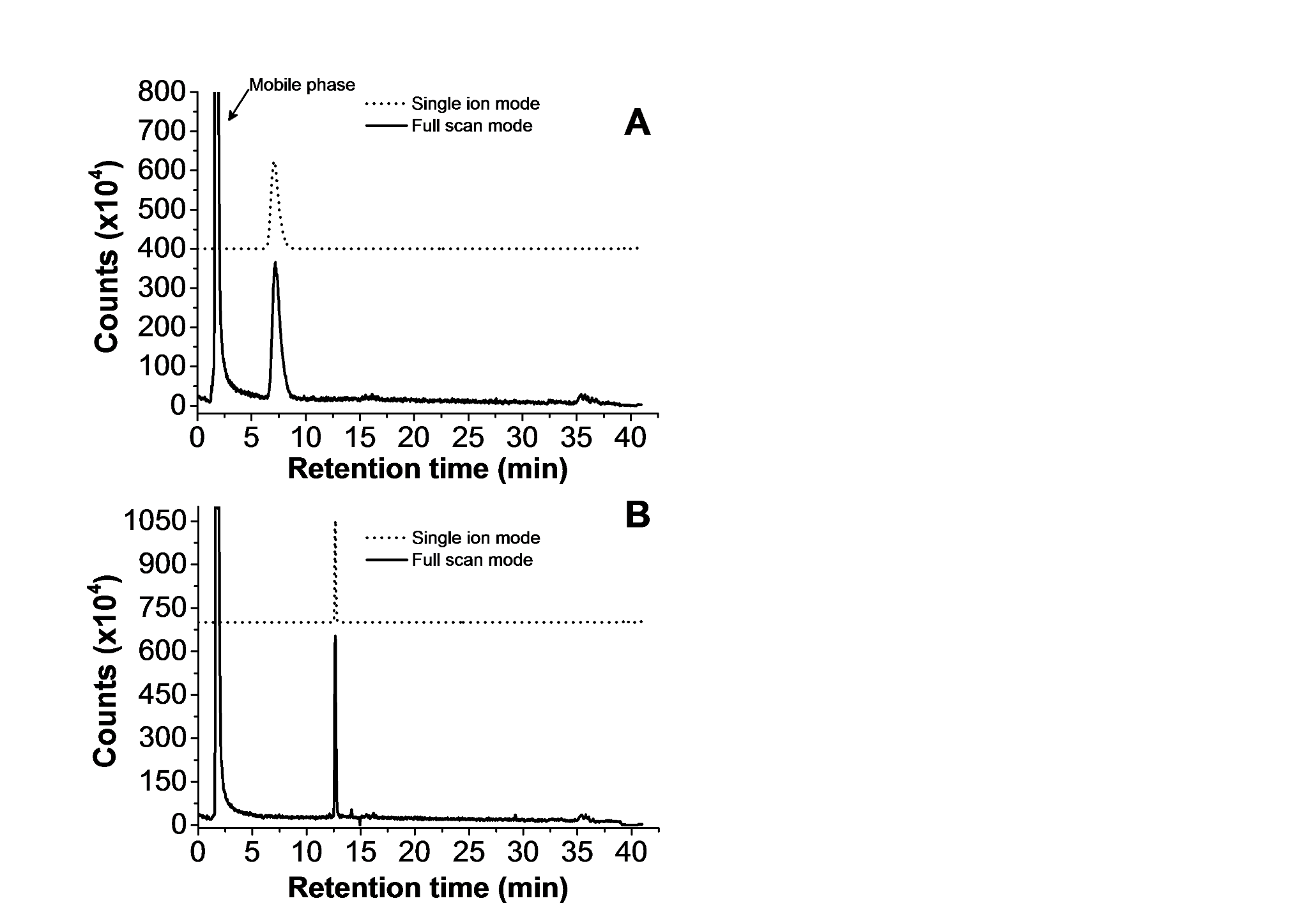
 **Supplementary S6.**

**Supplementary S7.**

**
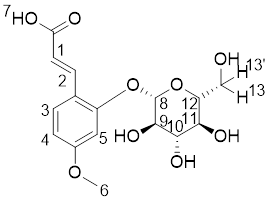
 A B**

**
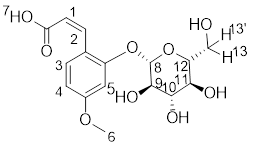
**

**
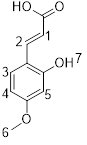
**

**C**

**Supplementary S8.**

**
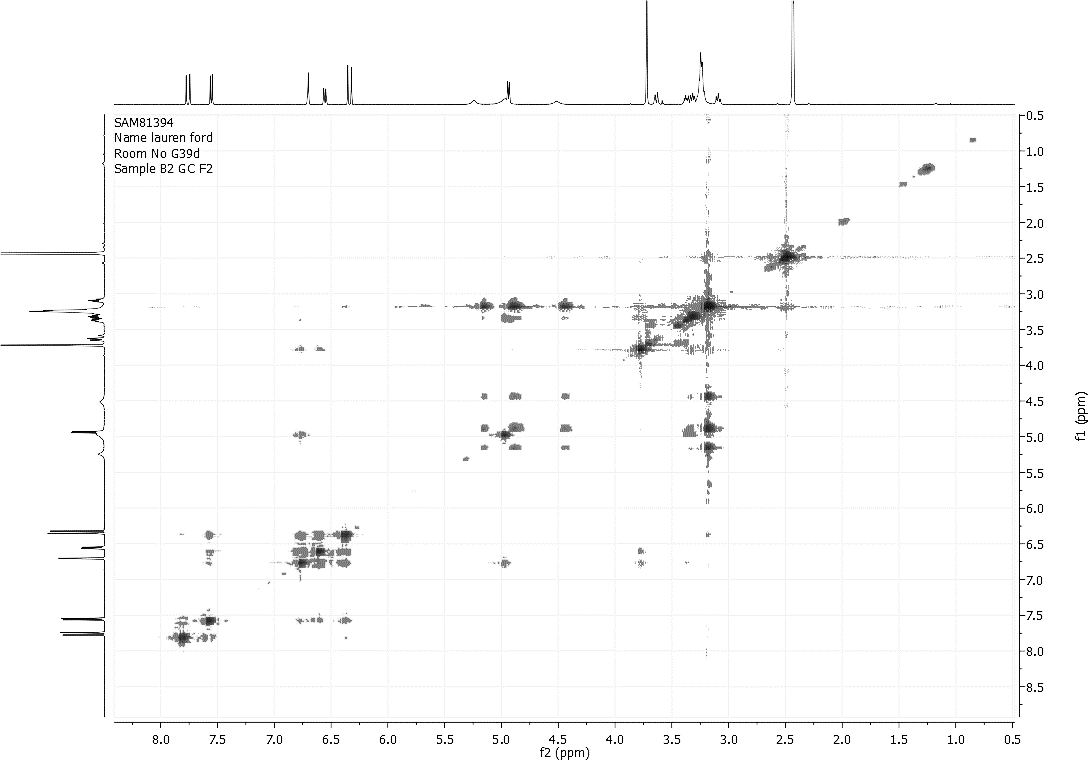
**

**Supplementary S9.**

**Supplementary S10.**


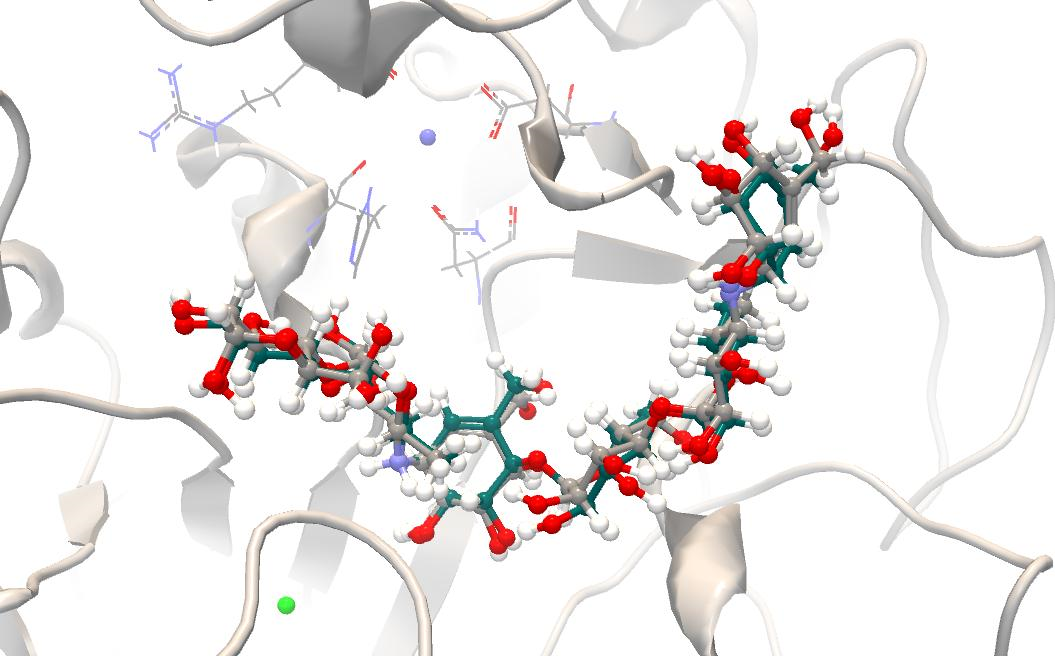

Supplement: Supplementary file 1 — Supplementary figures [file 41598_2018_23736_MOESM1_ESM.docx]
